# The Development of a Bilingual Interactive Video to Improve Physical Activity and Healthful Eating in a Head Start Population

**DOI:** 10.3390/ijerph111213065

**Published:** 2014-12-15

**Authors:** Veronica Piziak

**Affiliations:** Division of Endocrinology, Baylor Scott & White Health, College of Medicine, The Texas A&M Health Science Center, 2401 South 31st Street, Temple, TX 76508, USA; E-Mail: vpiziak@sw.org; Tel.: +1-254-215-0316; Fax: +1-254-215-0325

**Keywords:** preschool children, physical activity and nutrition, interactive videos

## Abstract

The prevalence of obesity in the Hispanic preschool population remains elevated, particularly among children in low income families below the poverty level. Obesity leads to the early onset of metabolic syndrome and Type 2 diabetes. The Head Start population of Texas is largely comprised of this high risk group. Their physical activity level is suboptimal in part due to lack of available outside play areas and time spent watching television and playing sedentary video games. Dietary intake is frequently high in sugar sweetened beverages and low in vegetables. The group is frequently bilingual with limited vocabulary and has not learned to read. Preserving their Mexican American culture is a concern. This article describes the development and assessment of a group of bilingual interactive video interventions to improve age appropriate physical activity while providing basic nutrition education focusing on increasing vegetable and water intake and decreasing sugar sweetened beverages. Suggestions for development and assessment of content were provided by focus groups of Head Start teachers, managers and dietitians in the Texas counties of Bastrop, Hidalgo and McLennon. A demonstration of the videos was conducted in Bastrop County. Teachers, students and managers felt that the videos provided excellent information, improved exercise participation and engaged the children.

## 1. Introduction 

The prevalence of overweight and obesity among United States preschool children age 2–5 years has decreased from 13.5% in 2003–2004 to 8.4% in 2011–2012; however, Mexican American children far exceeded the average level for obesity at 18.2% for boys and 15.2% for girls [1]. Hispanic preschool children from low income families are particularly affected with a prevalence of obesity in a study of the New York Special Supplemental Nutrition Program for Women, Infants and Children (WIC) population of 19.9% [2] and a study of Head Start children in Texas of 20% [3]. Obesity and ethnicity in Mexican American children markedly increases the risk of Type 2 diabetes and metabolic syndrome. In the study of Weiss *et al*. [4] where obese and nonobese children were compared for insulin resistance, hypertension, lipids levels, and the presence of metabolic syndrome, a preschool age group was included and 23.7% of the children were Hispanic. All of the risk factors increased with increasing obesity. Development of obesity in the Mexican American preschool child is multifactorial. Decreased physical activity can start in early childhood when safe, accessible outside play areas are unavailable. The shift to indoor activities leads to increased screen time as TV watching and computer time replace exercise [5]. Screen time and exercise time has been studied in this population and screen time exceeds the two hours a day recommended by the American Academy of Pediatrics in over 60% of the children on weekdays and 77% of the children on weekends. Over 80% of the children exercise over 30 min daily, but that is far from the American Academy of Pediatrics guideline of 120 min a day for this age group [6]. Dietary habits are also important contributors to the prevalence of obesity in our target group. Consumption of sugar and fructose sweetened beverages has also been linked to obesity in preschool children and the development of Type 2 diabetes and metabolic syndrome in adults [7]. The diets consumed by preschool children in the lower Rio Grande Valley and across the border in Mexico were studied [8] and far more carbohydrate and concentrated sweets were consumed by this population than is recommended by the United States Department of Agriculture (USDA). There was also low vegetable and fruit consumption in this population [9].

Targeting both the exercise and nutritional aspects of the problem of obesity early on, when children are establishing their eating and exercise patterns, is important. Head Start is a United States government funded locally operated preschool program providing basic education for children of low income families. Most of the children are from 3–5 years of age. The Head Start setting is ideally structured to provide both exercise and nutrition education options that are participatory, age appropriate, and culturally relevant. The purpose of this article is to describe the development and evaluation of a bilingual interactive video providing exercise and nutrition education for a Head Start population. The video combining nutrition education with exercise is novel. It is a link to our previous nutritional intervention allowing a review of the most important aspects of the previous nutritional education during exercise and allows the children to discuss nutrition during their rest breaks in the video so there is continuing focus on healthy living being a combination of nutrition and exercise. 

## 2. Methods 

### 2.1. Description of Videos and Related Equipment 

The objectives of the nutrition education portion are to decrease the consumption of sugar containing beverages, and increase fruit, vegetable, water and milk intake. The nutrition education section of the video builds on the bilingual pictorial nutrition “bingo” game described previously in this journal [9] and used by the Head Start facilities whose teachers and managers acted as the focus groups for evaluation of the videos. The exercise portion of the videos provides up to 10 min of vigorous exercise interspersed with exercises to improve fine motor skills. The narrative was composed by the author and animation was done by a theater arts student at Texas A&M University. The videos feature common cartoon animals: cows, goats, chickens, and horses. The animals ask the children to assist them in obtaining food appropriate for each animal. The animals and the children discuss appropriate food choices for the children and the animals while the children work to bring food from the countryside for the animals. The children provide enough food for each animal to be satisfied and the animals thank them for their efforts and remind them of the basic nutrition goals. The children are told that they have succeeded in helping the animals. Teachers were instructed in how to use the videos most effectively. Teacher participation in the exercises was encouraged. The teacher can pause the video for the children to prolong a particular exercise, rest or to discuss the nutrition points. The audio portion is bilingual. A sample of the videos featuring the bovine couple is provided in the Supplementary Material as video snippets. 

**Figure 1:**
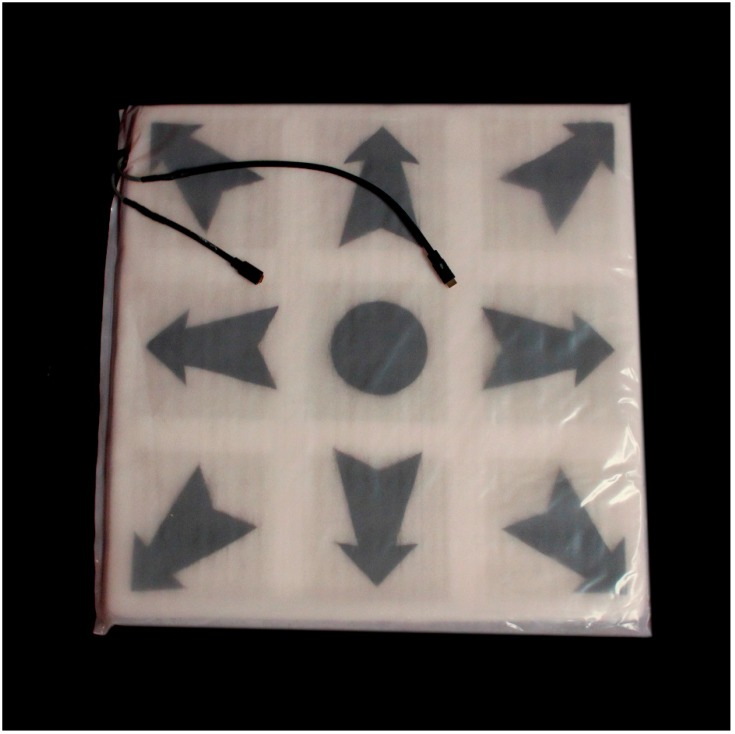
The exercise pad used in the intervention.

The Spanish dialog for the audio portions of the discs was translated and approved by the director of the Scott & White Spanish interpreter service and read by the interpreters and English dialog was approved and read by the author. The Scott & White audio division recorded the sound tracks. There is also one audio only exercise using dogs, to test comprehension without the visual stimulus. Both the video and audio discs may be used with computerized exercise pads that record the amount and level of exercise of the child using the pad. These are sent to a computer program which stores the information so that a comparison can be made for the group at the beginning and end of the study. The dance pads can also be used to look at individual performance. The pad has lights that flash when the child moves. The lights in the pads also provide motivation for the children to increase their participation. This exercise pad is shown in Figure 1 and was developed for the project by the Nanohmics Corporation (Austin, TX, USA). 

### 2.2. Focus Group Composition and Pilot Population 

The project was approved by the Scott and White Institutional Review Board. The teachers and managers who formed the focus groups were >75 % Hispanic and fluent in English and Spanish. The focus groups lasted an average of 1–2 h. The concept of the videos was initially discussed with 25 managers and teachers and the dietitian of the Bastrop County Head Start and 10 teachers and the manager of Hidalgo County Head Start who formed the first focus groups for development of the interactive video. 

The first video was demonstrated for the Bastrop and McLennon County focus groups consisting of teachers and managers and teachers aids. Each group contained 30–40 people and was tasked with testing the usability of the game for the preschool population. The first pilot video was then tested with 20 Head Start children from Bastrop county where their teachers and audiovisual assistant, as well as a representative from the Nanohmics corporation, and the author were present to assess the reaction of the children who were the target audience and identify technical problems. The group felt that the teachers were able to adequately evaluate the response of the children. They work with the same group of children for long periods of time and get to know them well. 

Revisions were made and the final focus group was from Bastrop County consisting of a manager and two teachers and the audiovisual assistant to evaluate the games for ease of incorporation into the Head Start educational day.

## 3. Results

The focus groups were set up to follow the steps suggested by Barnowski *et al*. for the development of games for behavioral change: concept development, demonstration of usability, use with the target audience, and identifying technical problems [10]. The results of the focus groups are shown in Table 1, Table 2, Table 3 and Table 4.

**Table 1:** Focus group 1 concept development.

**Suggestions for the Game (Concept)**
Videos must be bilingual due to limited English ability in some students. The focus groups for concept development felt that the bilingual aspect of the videos would play a part in preserving the Mexican American culture.
Cartoon characters must be familiar animals and be in pairs of different genders to help both the boys and girls identify with them.
Incorporate Exercises to improve fine motor skills.
Nutrition education section should incorporate bilingual bingo game rhymes since they impart the major nutritional goals and are known to many of the children.
**Quotations from Group Discussions**
Some of the younger children do not understand English well so the videos need to be bilingual.
Yes, bilingual videos might help the children learn English and also be helpful if the videos were used with the parents who are not fluent in English.
The children would benefit from a review of the nutrition concepts.

**Table 2:** Focus groups 2 demonstration video for teachers.

**Positive Comments/Suggestions**
Both groups felt that the video was understandable in either English or Spanish. These focus groups also felt that the bilingual nature of the videos would allow the children to continue using their Spanish.
Animals were appropriate and engaging.
Exercise was age appropriate.
Foods mentioned were familiar to the children.
The animals and situations were familiar to the children and are a part of their culture. A significant percentage of the parents are migratory farm workers harvesting fruits and vegetables and that occupation is portrayed as an important contribution to the society in the videos.
**Negative Comments/Suggestions**
Audio only disc not as effective, comprehension of the instructions by the children would be diminished because of lack of visual stimulation.
The suggestions were incorporate into the final videos and they were tested with children.
**Quotations from Group Discussions**
The foods mentioned are familiar to the children from the foods we serve at Head Start and the nutrition game we used last year.
The children really like moving to the videos, they are animated and the exercise intensity is greater than on the playground.
The audio only disc does not motivate the children. They don’t identify with the characters.

**Table 3:** Pilot video with the Head Start children.

**Positive Comments**
Children were animated and followed instruction for exercise well, participated enthusiastically and were able to keep pace with the video.
Children enjoyed the dance pads and were very animated.
All children participated in vigorous exercise and exercises to improve fine motor skills.
Children were immersed in the cartoon story.
Children participated in the nutrition game and we able answer some of the questions about foods.
More teacher education about the use of the video would help enhance the experience of the children.
**Negative Comments/Suggestions**
Pauses should be built in for the children to rest and for interaction about the nutrition points.
Plastic food models for the children to move would facilitate fine motor skills.
Dance pads connections were felt to be too long and present a hazard.
Set up of dance pads was time consuming.
**Quotations from Focus Groups**
All the children participated, some were in better physical shape than others.
The children really identified with the cartoon characters.
The dance pad cables were too long and I am afraid that the children will trip over them.

**Table 4:** Focus group for ease of use for the Head Start centers.

**Positive Comments**
Videos excellent and fit for the age group.
Characters engaging and there were no frightening aspects.
Excellent exercise for rainy days.
**Negative Comments**
Time for assembly for assemble of the dance pads was ten minutes but that was considered too long to be practical for the Head Start setting.
**Quotations from Focus Group**
The children will really like them. They will be great for rainy days.
There were no scary parts, That is great.
They will take too long to set up, they will have to stay connected in the storage area.

The videos were well received and it was felt that they would be useful for classroom teaching particularly on days when outdoor play was not possible. As a result of the demonstration pilot, the dance pads were reconfigured with shorter cables and simplified connections.

Although the children were not surveyed, we felt that the teachers were able to adequately evaluate the response of the children. They work with the same group of children for long periods of time and get to know them well. The teachers reported that the children were not only attentive but were more active than in their usual play and added their own movements to those of the animals. They looked forward to the video interaction.

## 4. Discussion

The study population is at risk for not only obesity, but the hypertension, diabetes, metabolic syndrome that are consequences of excess weight. If they are obese in childhood, they are likely to become obese adults. The economic status of their families increases the risk further. Preschool is an ideal time to intervene to prevent obesity since at this age they are developing their food and exercise preferences [11]. The children spend several hours on week days at the Head Start facility, have both structured and unstructured play time and eat meals and snacks there. Foods provided in the Head Start setting follow USDA nutritional guidelines for the type and amount of food provided to the children.

Games are an accepted format for interventions to promote nutrition and physical activity for both obesity prevention and intervention. Educational, informational games reinforce health eating principles that will hopefully provide skills to improve future food choices. Likewise, exercise instruction as an interactive game can allow children to learn that exercise should be a part of everyday. Games are an accepted format for obesity prevention and intervention and recently interactive games and some with video components for the preschool population have been reviewed. Mikkelsen *et al*. reviewed nutritional interventions some coupled with physical activity [12]. Most were educational sessions with limited interaction, but four of the 26 studies involved interactive games. The content emphasized increased intake of fruits and vegetables and most showed an improvement after the intervention. Although the long term outcomes on improving healthy eating behaviors are unknown the authors felt that the preschool setting had potential for long term habit formation and should be utilized [12]. Nixon *et al*. reviewed preschool based obesity prevention interventions including games based interventions [13]. The content included information about fruit, vegetable and water intake and well as movement skill development and strategies to decrease sedentary behavior. These authors felt that intervention that provided opportunities for skill development or success were well received. The majority of the studies did not have a significant long term impact on weight or dietary behavior [13]. Health Videogames were utilized in mainly school age children as reviewed by Lu *et al*.: five of the studies featured health education games, two focused on both nutrition and physical activity and two contained narratives. In the younger age group although most of the studies showed an increase in physical activity the change in obesity related outcomes such as BMI was only partially significant in one study or not significant in three studies [14]. Ickes *et al*. reviewed school based obesity interventions primarily in low income elementary schools in the United States. Nutritional education and increased physical activity were emphasized [15]. Five of the studies utilized health related games and all reported improvement in physical performance or significant changes in food choices. Four reported favorable changes in BMI [15]. Although long term outcomes of these interventions are unknown all of the authors felt that even short term improvement was encouraging. 

The interactive game in this study developed for the Head Start groups incorporates the recommendations of Ickes *et al*. for the development of school based obesity interventions [15]: it is tailored to the level of comprehension, income strata and culture of the target audience, with familiar characters and language, incorporates a combination of physical activity and nutrition education, the teachers received education before the demonstration and it is easily reproducible so that copies may be used at home to involve the parents. The game is also a narrative with a plot and characters and was developed to provide story immersion as suggested by Lu *et al*. [16]. The focus groups felt that story immersion was accomplished. There are personal movement experiences for the children, hopping, throwing, picking up small objects for example which are familiar, the characters engender affection, and there was suspension of disbelief. The weakness of this project was the failure of the dance pad recording devices to be workable for the teachers due to the set up time. Thus the individual child’s exercise progress could not be recorded during the demonstration period. This problem will be solved by providing funding to have the audiovisual assistant set up the dance pads and relieve the teachers of that responsibility. Overall, the teachers felt that the children were emotionally involved, motivated not only to help the animals eat well but also to eat well themselves. The teachers also felt that the children were satisfied that their tasks effectively helped the animals.

Presently, partners in Head Start areas are being sought to use the game. Parental involvement is critical if long term changes are to be achieved. When the educational intervention is put in place at a Head Start center, the parents are invited to participate. Parents periodically attend education programs about Head Start activities. When the nutrition game [9] was implemented, parents were invited to a demonstration where they played the game and were educated about the importance of proper nutrition and ways to shop to provide affordable nutritious food. A similar education program will be used for the exercise program. The parents were willing to change the food choices at home and hopefully, they will be able to take the time to exercise with their children when the program is set up. 

## 5. Conclusions

The problem of obesity in preschool children is significant and this study describes an engaging educational video which is satisfactory as judged by the teachers and fun for the children. Hopefully, some interested partners can be found in the future to conduct a larger scale study.

## Supplementary Files

Supplementary File 1
